# Study on the reform effect of payment based on curative effect value for TCM dominant diseases in China: an interrupted time series analysis

**DOI:** 10.3389/fpubh.2026.1818080

**Published:** 2026-07-02

**Authors:** Xiaoyan Xu, Tian Shen, Zhihui Li, Yuting Tao, Jiale Fang, Qiuyue Shen, Qiong Wu

**Affiliations:** 1 College of Humanities and Management, Hunan University of Chinese Medicine, Changsha, Hunan, China; 2 School of Public Health, Hangzhou Medical College, Hangzhou, Zhejiang, China

**Keywords:** anorectal diseases, diagnosis-related group, interrupted time series analysis, TCM-dominant diseases, value-based payment

## Abstract

**Background:**

Anorectal diseases, as prevalent and recurrent conditions for which Traditional Chinese Medicine (TCM) demonstrates distinct advantages, are closely associated with patients’ quality of life. The current Diagnosis-Related Group (DRG) payment system struggles to fully capture the unique value of TCM, whereas Value-Based payment models grounded in therapeutic efficacy offered a viable pathway for reforming payment mechanisms for TCM-dominant diseases.

**Methods:**

This study included a total of 5,304 patients diagnosed with hemorrhoids, anal fistulas, and other anorectal diseases who were admitted to the Proctology Department of a tertiary hospital in Hunan Province from August 2022 to August 2024. Interrupted Time Series (ITS) analysis was conducted, featuring two intervention points: January 2023 (DRG reform) and September 2023 (Value-Based payment reform). Nine indicators spanning three dimensions—Resource consumption (average inpatient cost per case, average length of stay), Cost structure (average TCM preparation cost per case, average TCM drug cost per case, average TCM diagnostic and treatment cost per case) and TCM characteristics (proportion of preparation cost in total drug revenue, proportion of TCM drug cost in total drug revenue, proportion of TCM diagnostic and treatment cost, proportion of discharged patients treated mainly with TCM)—were analyzed to evaluate both short-term and long-term effects of the dual policies, supplemented by age subgroup analysis.

**Results:**

After DRG reform, average inpatient cost per case (instantaneous decrease of CNY 1,131.27, *p* = 0.007) and average length of stay (instantaneous decrease of 1.69 days, *p* = 0.022) significantly decreased. However, average TCM drug cost per case exhibited an upward trend (an increase of CNY 19.46 per half-month, *p* < 0.001), and the proportion of discharged patients treated mainly with TCM decreased (a 2.16% reduction per month, *p* = 0.006) Following the implementation of Value-Based payment, average inpatient cost per case decreased instantaneously by CNY 910.82 (*p* = 0.006), and the average length of stay decreased instantaneously by 1.30 days (*p* = 0.024). Notably, the average TCM diagnostic and treatment cost per case increased significantly (an increase of CNY 100.21 every half month, *p* < 0.001); the proportion of TCM diagnostic and treatment cost in total revenue rose (an increase of 0.73% every half month, *p* < 0.001); and the proportion of discharged patients treated mainly with TCM increased instantaneously by 22.23% (*p* < 0.001). Subgroup analysis showed that the group under 60 years of age had a more significant response to the policies.

**Conclusion:**

Under the DRG framework, the Value-Based payment reform tied to therapeutic efficacy can effectively curb medical costs while incentivizing the application of TCM-specific services. Nevertheless, sustained monitoring is needed to evaluate its long-term impacts, particularly regarding age-related disparities in outcomes. These findings offer insights for refining TCM-inclusive medical insurance reimbursement policies.

## 1 Introduction

As China continues to deepen reforms on its medical insurance system, it has become a key national policy to formulate a medical insurance payment framework that fits the inherent principles of Traditional Chinese Medicine (TCM). The policy focuses prominently on supporting TCM inheritance and innovation, with particular emphasis on TCM-dominant conditions. TCM-dominant diseases refer to a category of conditions in which TCM techniques serve as the primary or key intervention during diagnosis and treatment—whether for the disease itself or a specific stage or aspect thereof. These conditions are underpinned by the TCM theoretical framework and supported by relatively mature diagnostic and treatment protocols. They demonstrate proven efficacy in key clinical outcomes, offer good cost-effectiveness, and are widely recognized by both patients and healthcare professionals. Furthermore, information regarding disease codes, clinical pathways, and cost structures is relatively standardized, facilitating their inclusion in Diagnosis-Related Group (DRG) payment management (1). Anorectal diseases, recognized as a condition in which TCM excels (2), are characterized by high prevalence and a tendency to recur, encompassing mixed hemorrhoids, anal fissures, anal fistulas, and rectal prolapse (3). The overall prevalence of anorectal diseases in China is as high as 50–60%, with mixed hemorrhoids accounting for the highest proportion (4). The rate of surgical intervention is rising yearly; issues such as postoperative pain and frequent complications exacerbate the strain on medical resources and severely impact patients’ quality of life, whilst the patient population is trending toward younger age groups. TCM emphasizes the combination of holistic regulation and local intervention. TCM advocates the combination of holistic regulation and localized intervention. Characteristic TCM therapies, including external herbal application, acupuncture, and thread suspension therapy, can effectively reduce tissue trauma and facilitate postoperative recovery. On this basis, a unique TCM diagnosis and treatment system that combines internal and external therapy to address both symptoms and root causes has been formed (5–7).

Diagnosis-Related Groups (DRG) originated in the United States as a case classification payment system anchored in medical resource consumption and have since been adopted across multiple countries (8–10). Since its introduction to China in 2009, continuous exploration has been conducted, yielding significant reform outcomes (11, 12). DRG payment helps standardize medical behaviors, reduce unnecessary medical services, thereby promoting the rational allocation of medical resources, improving the efficiency of medical services, and alleviating the economic burden on patients (13, 14). However, the DRG payment and evaluation system is primarily based on Western medical diagnoses, which are incompatible with the principles of TCM pattern differentiation. Consequently, it fails to reflect the characteristics of TCM, which places great emphasis on human resources, experience and technical expertise, leading to the undervaluation of TCM’s distinctive value at the payment stage (15). Since 2019, China has issued a series of policies, including the Guiding Opinions on Medical Insurance Supporting the Inheritance and Innovative Development of Traditional Chinese Medicine and the 14th Five-Year Plan for the Development of Traditional Chinese Medicine. These policies explicitly call for the screening of TCM-dominant conditions, the exploration of a TCM-adapted medical insurance payment system, and the implementation of equal payment for equivalent clinical outcomes for the same disease treated by either TCM or Western medicine (16). As a National Comprehensive Reform Demonstration Zone for TCM, the Hunan Provincial Medical Insurance Bureau issued the Notice on Implementing Value-Based Payment for TCM-Dominant Conditions within Regional DRG Payment (Trial) in July 2023 (17). The policy adopts Value-Based Payment in designated medical institutions, fully harnessing TCM’s unique clinical characteristics, technical value and price advantages. It promotes shared benefits for insured patients, medical institutions, and medical professionals, as well as the medical insurance fund, and improves the medical insurance payment system that accommodates the characteristics of TCM services.

Value-Based payment reform is a value-oriented medical insurance payment policy, which was first practiced in Liuzhou, Guangxi, China (18). The concept of “Value-Based healthcare” was first proposed by Porter from Harvard University in 2006 (19), who defined the value of medical services as “health outcomes per unit of medical input” (20). Practices based on Value-Based healthcare have become an important topic in global healthcare system reform. This framework stresses that value assessment should center on patients and their medical conditions. Relevant outcome indicators include clinical parameters, functional performance, rehabilitation progress and long-term health sustainability (19). It is highly consistent with the philosophy of TCM diagnosis and treatment, which puts people first and focuses on the body’s functional status (21, 22). Anorectal diseases, as a type of common and frequently occurring diseases that seriously affect patients’ quality of life, involve not only clinical treatment effects but also patients’ rehabilitation process, quality of life, and psychological state. Therefore, applying the concept of Value-Based healthcare to the diagnosis and treatment of anorectal diseases is of great significance for giving full play to the advantages and value of TCM and establishing a sound medical insurance payment method in line with the characteristics of TCM.

At present, the demonstration of the value of TCM in China is still in its infancy, with existing research primarily focusing on three dimensions. First, studies have focused on the localized interpretation of Value-Based healthcare theory, largely exploring the theoretical justification of Value-Based payment for treatment outcomes from the perspectives of incentive compatibility and stakeholder interests (23–25). Second, research has evaluated outcomes at specific policy junctures, often using early pilot schemes in cities such as Liuzhou and Luzhou as case studies. These studies employ difference-in-differences (DID) or pre-post control designs to demonstrate the short-term effectiveness of this model in reducing per-case costs and shortening hospital stays (26–28). Third, existing causal identification studies adopt single economic indicators, which are mostly limited to macro revenue and expenditure metrics such as total costs and medical supplies expenses (29, 30). Insufficient attention has been paid to the incremental value of TCM-specific techniques under the DRG payment system. Additionally, few studies have investigated the synergistic effects and dynamic evolutionary rules of the combined implementation of DRG reform and Value-Based payment reform. To address these gaps, this study analyzed patients from the Anorectal Department of a tertiary hospital in Hunan Province as the research object, adopts Interrupted Time Series (ITS) analysis to conduct an in-depth analysis of the dual policy effects of Value-Based payment for TCM-dominant diseases under DRG payment. It aims to provide a scientific basis for deepening the reform of medical insurance payment in TCM hospitals, exploring medical insurance payment policies conducive to the inheritance, innovation, and development of TCM, and at the same time exploring innovative paths for the localized practice of Value-Based healthcare. Ultimately, it seeks to establish a dynamic equilibrium between controlling costs and ensuring curative effects.

## 2 Materials and methods

### 2.1 Value-based payment system

Firstly, the selection criteria for conditions included in the Value-Based payment scheme are as follows: conditions that demonstrate significant distinctive advantages of TCM, have clear treatment protocols, exhibit outstanding clinical efficacy, and have well-defined discharge criteria. These conditions are selected by the Provincial Medical Insurance Bureau in conjunction with the Provincial Administration of TCM, based on expert opinions, objective circumstances and settlement data. Secondly, eligible cases must fall within the clearly defined list of included conditions, have indications for surgery, and meet admission criteria. Furthermore, they must follow the clinical pathway for treatment and be discharged in accordance with efficacy evaluation standards to be included within the scope of Value-Based payment. The efficacy assessment integrates three categories of core outcome indicators: firstly, objective Western medical indicators, such as wound healing status, complication rates and readmission rates; secondly, TCM syndrome elements, such as dynamic changes in diagnostic dimensions like ‘damp-heat descending’ and ‘blood stasis and qi stagnation’; and thirdly, patient-reported outcomes, including pain scores, bowel function and activities of daily living, reflecting the multi-indicator integration approach advocated in the comprehensive evaluation of TCM clinical efficacy (31). The assessment results are directly linked to medical insurance payments: cases meeting the efficacy standards are reimbursed by multiplying the weighting of the corresponding surgical DRG group by a specified discount rate; cases that fail to meet efficacy standards, are transferred to Western medical surgery during treatment, or are readmitted for the same condition shortly after discharge and deemed to have failed TCM treatment, are excluded from the Value-Based payment mechanism and revert to standard DRG payment. Multidimensional outcome indicators, standardized clinical pathways, and admission/exclusion criteria collectively form a closed-loop system, aligning with the TCM value assessment framework that integrates disease and syndrome and is oriented toward multiple primary outcomes (32).

### 2.2 Study design

This study employed ITS to evaluate changes in medical resource consumption, medical cost structure, and TCM-specific indicators before and after the implementation of the Value-Based payment reform. The analytical window spanned August 2022 to August 2024, with two distinct policy intervention nodes: the launch of DRG payment reform in January 2023, and the implementation of Value-Based payment for TCM-dominant diseases in September 2023.

### 2.3 Data sources and sampling

This study selected a tertiary Grade-A Traditional Chinese Medicine hospital in Hunan Province as the study site. As the first tertiary Grade-A TCM hospital in Hunan Province and the provincial center for TCM medical treatment, education, and research, the hospital ranks among the advanced TCM hospitals in China in terms of TCM characteristics and comprehensive capacity building. As a designated institution for DRG medical insurance payment in Changsha, the hospital officially launched the DRG payment reform on January 1, 2023. To strengthen support for TCM under the DRG payment model, the Hunan Provincial Healthcare Security Bureau issued the “Notice on Implementing Value-Based Payment for TCM - Dominant Diseases in Regional DRG Payment (Trial)” in August 2023. This policy selected 22 TCM - dominant diseases with clear diagnoses, mature TCM diagnosis and treatment technologies, and clear indicators for evaluating curative effects to implement Value-Based payment reform, including mixed hemorrhoids, high anal fistula, and complex anal fistula. The Value-Based payment reform came into effect on 1 September 2023.

In this study, first-page data of medical records of patients in the Anorectal Department with primary diagnoses of mixed hemorrhoids, high anal fistula, and complex anal fistula were collected from August 2022 to August 2024, including personal basic information, disease information, and expense information, initially comprising 5,410 cases. Cases with logical abnormalities, duplicate or missing items, length of stay > 60 days, and inpatient expenses < CNY 5 were excluded, leaving 5,304 analytically valid records. With half-month as one cycle (50 time points in total), two intervention points were set: January 2023 was the intervention point for DRG payment, and September 2023 was the intervention point for Value-Based payment. The period from August 2022 to December 2022 was before the DRG reform; the period from January 2023 to August 2023 was after the DRG reform but before the implementation of Value-Based payment; and the period from September 2023 to August 2024 was after the implementation of Value-Based payment.

### 2.4 Study indicators

Based on literature review and expert consultation (33), nine indicators spanning three dimensions were selected as evaluation indicators for reform effects (Table 1) including: Resource consumption: average inpatient cost per case, average length of stay (LOS); Cost structure: average TCM preparation cost per case, average TCM drug cost per case, average TCM diagnosis and treatment cost per case; TCM characteristics: proportion of preparation cost in total drug revenue, proportion of TCM drug cost in total drug revenue, proportion of TCM diagnosis and treatment cost, proportion of discharged patients treated mainly with TCM.

**Table 1:** Evaluation dimensions and indicators.

Dimension	Indicator	Definition
Resource consumption	Average Inpatient Cost per Case	Total Inpatient Cost / Total Number of Discharged Patients
	Average Length of Stay (LOS)	Total Length of Hospital Stay / Total Number of Discharged Patients
Cost structure	Average TCM Preparation Cost per Case	Total TCM Preparation Cost / Total Number of Discharged Patients
	Average TCM Drug Cost per Case	Total TCM Drug Cost / Total Number of Discharged Patients
	Average TCM Diagnosis and Treatment Cost per Case	Total TCM Diagnosis and Treatment Cost / Total Number of Discharged Patients
TCM characteristics	Proportion of Preparation Cost in Total Drug Revenue	Total TCM Preparation Cost / Total Drug Cost
	Proportion of TCM Drug Cost in Total Drug Revenue	Total TCM Drug Cost / Total Drug Cost
	Proportion of TCM Diagnosis and Treatment Cost	Total TCM Diagnosis and Treatment Cost / Total Inpatient Cost
	Proportion of Discharged Patients Treated Mainly with TCM	Number of Discharged Patients Treated Mainly with TCM / Total Number of Discharged Patients

### 2.5 Data analysis methods

#### 2.5.1 Interrupted time series analysis

Interrupted Time Series (ITS) analysis evaluates policy effects by comparing changes in indicator trends using multi-time-point data before and after an intervention, and is recognized as the gold standard for assessing the longitudinal effects of policy interventions (34). Compared with traditional methods, ITS can accurately identify both the immediate effects and long-term trends of interventions without requiring a control group, making it uniquely advantageous in evaluating policy effects in fields such as public health and health management (35).

In this study, the implementation times of the DRG reform and the Value-Based payment reform in Changsha were set as intervention points. With time as the influencing factor, and resource consumption, cost structure, and TCM characteristics as outcome indicators, a linear regression model was established for analysis. The equation is as follows:

$\begin{array}{l} Y_{t}=\beta_{0}+\beta_{1}\times \text{time}+\beta_{2}\times \text{Interv}1+\beta_{3}\times \text{Timeafterinterv}1+\\ \;\beta_{4}\times \text{Interv}2+\beta_{5}\times \text{Timeafterinterv}2+\varepsilon_{t} \end{array}$

Independent parameters were introduced to capture the immediate level changes and long-term trend changes of the outcome variables caused by the two reforms, respectively. *Y_t_* represents the observed value of the dependent variable; *β_0_* is the initial state parameter, representing the estimated value of the dependent variable level before any intervention; *β_1_* represents the initial trend intensity before the first reform, indicating the estimated trend of the dependent variable before DRG implementation;time denotes the time series, with a half-months as one cycle (50 time points in total);*β_2_* is the estimated value of the instantaneous level change of the dependent variable after DRG reform implementation;*β_3_* represents the change in the trend of the dependent variable before and after DRG reform (i.e., the difference in slopes), and *β_1_ + β_3_* represents the trend in the independent variable following the implementation of the DRG reform and prior to the introduction of Value-Based payment; *β_4_* is the estimated value of the instantaneous level change of the dependent variable after the implementation of Value-Based payment; *β_5_* represents the change in the trend of the dependent variable before and after the implementation of Value-Based payment (i.e., the difference in slopes), and *β_1_* + *β_3_* + *β_5_* represents the trend in the variable following the implementation of Value-Based payment based on DRG reform; *ε_t_* is the random error term. In addition, the Durbin-Watson statistic was used to fit the Prais-Winsten estimation for addressing autocorrelation, and robust standard errors were adopted in the analysis (36, 37).

#### 2.5.2 Statistical analysis

Excel 2016 was used to organize the database, whilst Stata 18.0 was employed to perform the Kruskal-Wallis H test and ITS analysis. Frequencies and proportions were used to describe the demographic characteristics, clinical features and utilization of traditional Chinese medicine services at different stages of the reform. The Kruskal-Wallis H test was employed to compare differences in relevant indicators before and after the reform. The ITS analysis was used to assess trends in relevant indicators before and after the reform. Unless otherwise stated, a two-tailed *α* = 0.05 was used as the significance level for all analyses.

## 3 Results

### 3.1 Comparison of relevant indicators of anorectal patients before and after the reform

A total of 5,304 cases were included in this study, with 920 cases (17.3%) in the pre-DRG period, 4,384 cases (82.7%) in the post-DRG period, and 2,593 cases (48.9%) in the post-Value-Based payment period. Significant differences were observed in average inpatient cost per case, average length of stay, average TCM preparation cost per case, average TCM drug cost per case, average TCM diagnosis and treatment cost per case, proportion of preparation cost in total drug revenue, proportion of TCM drug cost in total drug revenue, proportion of TCM diagnosis and treatment cost, and proportion of discharged patients treated mainly with TCM across the three policy stages (Table 2).

**Table 2:** Comparison of differences in evaluation indicators before and after the reform.

Indicators	Before DRG reform (time = 10)	After DRG reform but before Value-Based payment for therapeutic effects (time = 16)	After Value-Based payment for therapeutic effects (time = 24)	Statistical value	*p*-value
Average inpatient cost per case	11595.83 ± 997.93	12335.48 ± 506.47	12588.52 ± 578.75	10.567	0.005
Average length of stay	12.40 ± 1.73	12.58 ± 0.93	11.89 ± 0.63	9.568	0.008
Average TCM preparation cost per case	699.47 ± 75.64	769.08 ± 35.22	826.69 ± 90.11	13.667	0.001
Average TCM drug cost per case	1370.62 ± 117.93	1542.81 ± 114.09	1655.18 ± 92.66	23.751	0.001
Average TCM diagnosis and treatment cost per case	1091.28 ± 114.29	1133.93 ± 70.23	1878.12 ± 915.09	25.216	<0.001
Proportion of preparation cost in total drug revenue (%)	23.13 ± 2.67	21.96 ± 1.49	26.14 ± 3.67	16.281	<0.001
Proportion of TCM drug cost in total drug revenue (%)	45.20 ± 2.95	43.99 ± 3.10	52.33 ± 5.42	28.071	<0.001
Proportion of TCM diagnosis and treatment cost (%)	9.42 ± 0.71	9.19 ± 0.41	14.75 ± 6.75	27.081	<0.001
Proportion of discharged patients treated mainly with TCM (%)	89.16 ± 8.88	79.30 ± 7.88	91.88 ± 5.16	18.564	<0.001

### 3.2 Interrupted time series analysis of resource consumption indicators

Upon implementing the DRG reform, we observed an immediate and statistically significant reduction in both cost and length of stay. Average inpatient cost per case dropped by CNY 1,131.27 (*p* = 0.007), and the average length of stay fell by 1.69 days (*p* = 0.022). Regarding the change in the trend before and after the reform, the *β_3_* value for the average inpatient cost per case was −191.05 (*p* = 0.002), while the *β_3_* value for the average length of stay was not statistically significant (*p* = 0.526). After the reform, the average inpatient cost per case increased by CNY 62.88 every half month on average (*p* = 0.020), and the average length of stay increased by 0.11 days every half month on average (*p* = 0.024).

**Figure 1:**
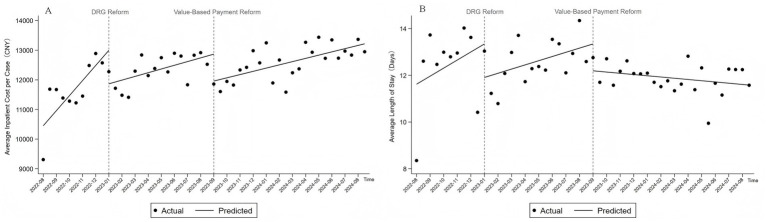
Interrupted time series trend chart of resource consumption indicators. Panel **A** is a scatter plot with a trend line showing average inpatient cost per case in CNY over time from August 2022 to August 2024, with vertical dashed lines marking DRG Reform and Value-Based Payment Reform. Panel **B** is a scatter plot with a trend line showing average length of stay in days over the same period, also marking DRG Reform and Value-Based Payment Reform. Both panels display actual and predicted values, with a legend indicating black dots as actual data and solid lines as predicted trends.

Similarly, after introducing Value-Based payment for therapeutic effects, there was another immediate decrease in resource utilization: the average inpatient cost per case fell by CNY 910.82 (*p* = 0.006), and the average length of stay decreased by 1.30 days (*p* = 0.024). For the change in the trend before and after the reform, the *β_5_* value for the average inpatient cost per case was not statistically significant, while the *β_5_* value for the average length of stay was −0.13 (*p* = 0.014). After the reform, the average inpatient cost per case showed an upward trend, increasing by CNY 54.76 every half month on average (*p* < 0.001), while the trend of the average length of stay was not statistically significant (Figure 1; Table 3).

**Table 3:** Results of interrupted time series (ITS) analysis for evaluation indicators.

Indicators	*β_0_*	*β_1_*	*β_2_*	*β_3_*	*β_4_*	*β_5_*	*β_1+_β_3_*	*β_1+_β_3+_β_5_*
Average inpatient cost per case	10455.49 (<0.001)	253.93 (<0.001)	−1131.27 (0.007)	−191.05 (0.002)	−910.82 (0.006)	−8.12 (0.785)	62.88 (0.020)	54.76 (<0.001)
Average length of stay	11.73 (<0.001)	0.17 (0.073)	−1.69 (0.022)	−0.07 (0.526)	−1.30 (0.024)	−0.13 (0.014)	0.11 (0.024)	−0.02 (0.320)
Average TCM preparation cost per case	647.11 (<0.001)	11.90 (0.049)	−16.39 (0.715)	−9.35 (0.159)	−75.18 (0.037)	7.13 (0.035)	2.54 (0.382)	9.68 (<0.001)
Average TCM drug cost per case	1214.51 (<0.001)	35.21 (<0.001)	−170.95 (0.004)	−15.75 (0.058)	−111.05 (0.014)	−14.34 (<0.001)	19.46 (<0.001)	5.12 (0.011)
Average TCM diagnosis and treatment cost per case	979.52 (0.007)	14.79 (0.795)	54.15 (0.872)	−26.49 (0.718)	−218.71 (0.481)	111.91 (0.010)	−11.70 (0.711)	100.21 (<0.001)
Proportion of preparation cost in total drug revenue (%)	24.40 (<0.001)	−0.69 (0.081)	7.23 (<0.001)	0.39 (0.452)	1.65 (0.372)	0.73 (0.025)	−0.30 (0.199)	0.43 (0.005)
Proportion of TCM drug cost in total drug revenue (%)	47.35 (<0.001)	−0.92 (0.175)	6.00 (0.058)	1.04 (0.255)	0.53 (0.858)	0.43 (0.457)	0.12 (0.761)	0.56 (0.046)
Proportion of TCM diagnosis and treatment cost (%)	9.68 (<0.001)	−0.11 (0.808)	1.06 (0.662)	−0.01 (0.980)	−0.90 (0.690)	0.85 (0.013)	−0.12 (0.626)	0.73 (<0.001)
Proportion of discharged patients treated mainly with TCM (%)	78.95 (<0.001)	2.23 (0.004)	−13.73 (0.014)	−3.31 (<0.001)	22.23 (<0.001)	1.04 (0.021)	−1.08 (0.006)	−0.04 (0.831)

### 3.3 Interrupted time series analysis of cost structure indicators

Upon implementing the DRG reform, there were no significant immediate changes in either average TCM preparation cost per case or average TCM diagnosis and treatment cost per case. However, we observed an immediate reduction in average TCM drug cost per case of CNY 170.95 (*p* = 0.004). The changes in the trends of the average TCM preparation cost per case, average TCM drug cost per case, and average TCM diagnosis and treatment cost per case before and after the reform were all not statistically significant. After the reform, the trends in the average TCM preparation cost per case and average TCM diagnosis and treatment cost per case were not statistically significant, while the average TCM drug cost per case showed an upward trend (*p* < 0.001), increasing by CNY 19.46 every half month on average.

**Figure 2:**
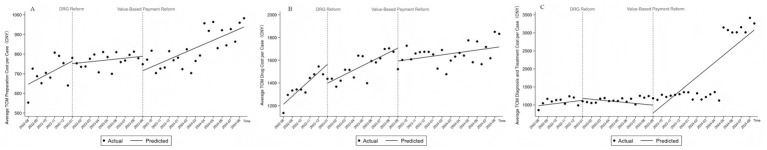
Interrupted time series trend chart of cost structure indicators. Three-panel line chart compares actual versus predicted average costs per case for TCM preparation **(A)**, TCM drug **(B)**, and TCM diagnosis and treatment **(C)** in CNY from August 2022 to August 2024, with markers for DRG Reform and Value-Based Payment Reform. Each panel shows fluctuating but generally rising trends, especially after reforms, with diagnosis and treatment costs showing the largest increase post-reforms.

Following the implementation of the Value-Based payment reform for therapeutic effects, several immediate level changes occurred: the average TCM preparation cost per case decreased instantaneously by CNY 75.18 (*p* = 0.037), the average TCM drug cost per case decreased instantaneously by CNY 111.05 (*p* = 0.014), and the instantaneous change in the average TCM diagnosis and treatment cost per case was not statistically significant. The changes in the trends of the average TCM preparation cost per case, average TCM drug cost per case, and average TCM diagnosis and treatment cost per case before and after the reform were all statistically significant (all *p* < 0.05), with *β_5_* values being CNY 7.13, CNY -14.34, and CNY 111.91, respectively. After the reform, the average TCM preparation cost per case, average TCM drug cost per case, and average TCM diagnosis and treatment cost per case all showed upward trends (all *p* < 0.05), increasing by CNY 9.68, CNY 5.12, and CNY 100.21 every half month on average, respectively, (Figure 2; Table 3).

### 3.4 Interrupted time series analysis of TCM characteristic indicators

Upon the implementation of the DRG reform, the instantaneous changes in the proportion of TCM drug cost in total drug revenue and the proportion of TCM diagnosis and treatment cost were not statistically significant (*p* > 0.05). The instantaneous change in the proportion of preparation cost in total drug revenue was 7.23% (*p* < 0.001), and the proportion of discharged patients treated mainly with TCM decreased instantaneously, with a *β_2_* value of −13.73 (*p* = 0.014). Regarding the changes in trends before and after the reform, the *β_3_* values for the proportion of preparation cost in total drug revenue, proportion of TCM drug cost in total drug revenue, and proportion of TCM diagnosis and treatment cost were all not statistically significant, while the *β_3_* value for the proportion of discharged patients treated mainly with TCM was −3.31 (*p* < 0.001). After the reform, the proportion of discharged patients treated mainly with TCM decreased by an average of 1.08% every half month (*p* = 0.006), while the trends in the proportion of preparation cost in total drug revenue, proportion of TCM drug cost in total drug revenue, and proportion of TCM diagnosis and treatment cost were not statistically significant.

**Figure 3:**
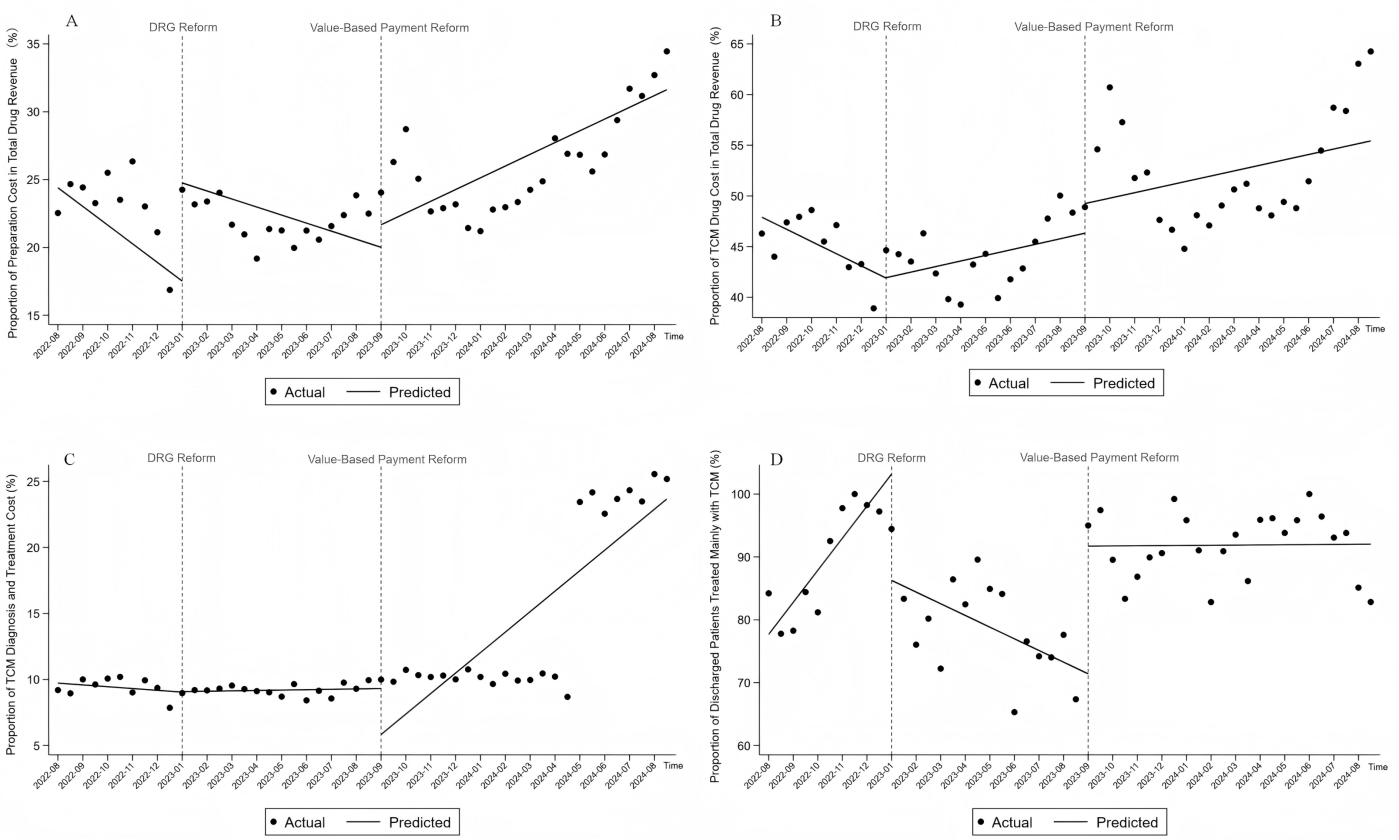
Interrupted time series trend chart of TCM characteristic indicators. Four line and scatter plots labeled **(A–D)**, each showing actual versus predicted values over time from August 2022 to August 2024, with vertical dashed lines indicating DRG Reform and Value-Based Payment Reform. **(A)** shows the proportion of preparation cost in total drug revenue, **(B)** shows the proportion of TCM drug cost in total drug revenue, **(C)** shows the proportion of TCM diagnosis and treatment cost, and **(D)** shows the proportion of discharged patients treated mainly with TCM. Trends shift at reform points, with both increases and decreases observed across the plots.

Upon the implementation of the Value-Based payment reform for therapeutic effects, the instantaneous changes in the proportion of preparation cost in total drug revenue, proportion of TCM drug cost in total drug revenue, and proportion of TCM diagnosis and treatment cost were not statistically significant, but the proportion of discharged patients treated mainly with TCM increased instantaneously by 22.23% (*p* < 0.001). For the changes in trends before and after the reform, the trend changes in the proportion of preparation cost in total drug revenue, proportion of TCM diagnosis and treatment cost, and proportion of discharged patients treated mainly with TCM were all statistically significant (*p* < 0.05), with values of 0.73, 0.85, and 1.04% per half month respectively, while the proportion of TCM drug cost in total drug revenue showed no statistical significance. After the reform, the proportion of preparation cost in total drug revenue, proportion of TCM drug cost in total drug revenue, and proportion of TCM diagnosis and treatment cost all increased (*p* < 0.05), with average increases of 0.43, 0.56, and 0.73% every half month, respectively. The trend in the proportion of discharged patients treated mainly with TCM was not statistically significant (*p* > 0.05) (Figure 3; Table 3).

### 3.5 Interrupted time series analysis of evaluation indicators by age subgroup

To further explore the impact of the Value-Based payment reform for therapeutic effects among different age groups, this study categorized patients into subgroups aged under 60 years of age and aged 60 years and above, and conducted a subgroup analysis of the reform effects by age via the ITS analysis.

#### 3.5.1 Interrupted time series analysis of resource consumption indicators by age subgroup

**Figure 4:**
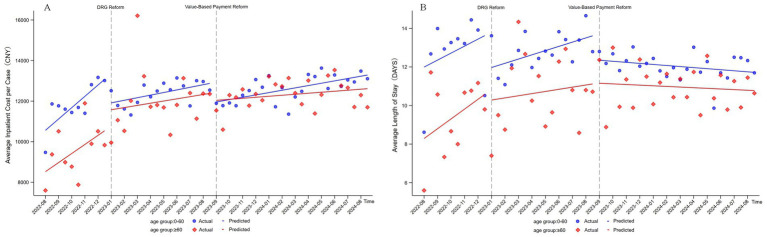
Interrupted time series trend chart of resource consumption indicators by age subgroup. Two-panel line and scatter plot comparing time trends before and after DRG Reform and Value-Based Payment Reform. Panel **A** shows average inpatient cost per case; panel **B** shows average length of stay. Blue circles and lines represent age group under sixty, red diamonds and lines represent age group sixty and above. Both actual and predicted values are plotted, with two vertical dashed lines indicating reform points along the time axis from August 2022 to August 2024.

In the group under 60 years of age, upon the implementation of the DRG reform, the average inpatient cost per case decreased instantaneously by CNY 1,439.24 (*p* = 0.003), and the average length of stay decreased instantaneously by 1.83 days (*p* = 0.019). The trend change in average inpatient cost per case before and after the reform (*β_3_*) was CNY -215.13 (*p* = 0.002). After the reform, the average inpatient cost per case increased by an average of CNY 63.99 every half month (*p* = 0.036), and the average length of stay increased by an average of 0.11 days every half month (*p* = 0.027). Upon the implementation of the Value-Based payment reform for therapeutic effects, the average inpatient cost per case decreased instantaneously by CNY 977.62 (*p* = 0.009), and the average length of stay decreased instantaneously by 1.37 days (*p* = 0.023). After the reform, the average inpatient cost per case increased by CNY 57.69 every half month on average (*p* = 0.001), exhibiting a slower growth rate compared to the period after the DRG reform. However, in the group aged 60 years and above, no statistically significant changes were observed in average inpatient cost per case or average length of stay before and after the reforms (all *p* > 0.05; Figure 4; Table 4).

**Table 4:** Results of interrupted time series (ITS) analysis for evaluation indicators by age subgroup.

Indicators	*β_0_*	*β_1_*	*β_2_*	*β_3_*	*β_4_*	*β_5_*	*β_1+_β_3_*	*β_1+_β_3+_β_5_*
Average inpatient cost per case								
Aged <60 years	10561.64 (<0.001)	279.12 (<0.001)	−1439.24 (0.003)	−215.13 (0.002)	−977.62 (0.009)	−6.30 (0.001)	63.99 (0.036)	57.69 (0.001)
Aged ≥60 years	8523.14 (<0.001)	223.67 (0.106)	815.39 (0.424)	−169.98 (0.278)	−394.10 (0.632)	−28.77 (0.718)	53.69 (0.436)	24.92 (0.512)
Average length of stay								
Aged <60 years	11.99 (<0.001)	0 0.18 (0.074)	−1.83 (0.019)	−0.07 (0.515)	−1.37 (0.023)	−0.14 (0.015)	0.11 (0.027)	−0.03 (0.297)
Aged ≥60 years	8.29 (<0.001)	0.25 (0.128)	−0.55 (0.663)	−0.20 (0.281)	−0.02 (0.985)	−0.07 (0.432)	0.06 (0.492)	−0.02 (0.706)
Average TCM preparation cost per case								
Aged <60 years	660.45 (<0.001)	11.22 (0.092)	−15.37 (0.757)	−9.02 (0.219)	−81.06 (0.042)	7.99 (0.033)	2.20 (0.495)	10.19 (<0.001)
Aged ≥60 years	484.86 (<0.001)	21.17 (0.075)	−9.44 (0.918)	−15.51 (0.235)	−26.28 (0.705)	0.11 (0.987)	5.66 (0.324)	5.76 (0.067)
Average TCM drug cost per case								
Aged <60 years	1219.95 (<0.001)	37.59 (<0.001)	−200.96 (0.002)	−18.40 (0.040)	−121.63 (0.012)	−13.30 (0.004)	19.19 (<0.001)	5.89 (0.007)
Aged ≥60 years	1100.98 (<0.001)	25.95 (0.087)	23.65 (0.835)	−1.02 (0.951)	−81.30 (0.358)	−26.30 (0.002)	24.94 (0.001)	−1.36 (0.727)
Average TCM diagnosis and treatment cost per case								
Aged <60 years	983.44 (0.008)	17.59 (0.763)	51.43 (0.881)	−30.40 (0.686)	−242.12 (0.447)	117.35 (0.008)	−12.81 (0.692)	104.54 (<0.001)
Aged ≥60 years	12904.33 (0.002)	−550.43 (0.431)	2626.48 (0.579)	431.76 (0.612)	−3242.36 (0.433)	821.17 (0.077)	−118.67 (0.748)	702.50 (0.002)
Proportion of preparation cost in total drug revenue								
Aged <60 years	22.49 (<0.001)	−0.81 (0.082)	8.81 (<0.001)	0.43 (0.484)	1.51 (0.487)	0.77 (0.045)	−0.38 (0.167)	0.39 (0.030)
Aged ≥60 years	1.40 (0.013)	0.11 (0.283)	−0.09 (0.902)	−0.10 (0.363)	0.43 (0.482)	0.06 (0.332)	0.01 (0.923)	0.06 (0.031)
Proportion of TCM drug cost in total drug revenue								
Aged <60 years	43.12 (<0.001)	−1.00 (0.199)	7.22 (0.043)	0.94 (0.373)	1.02 (0.760)	0.56 (0.405)	−0.06 (0.900)	0.51 (0.119)
Aged ≥60 years	3.18 (0.005)	0.15 (0.454)	0.25 (0.867)	−0.11 (0.625)	1.09 (0.368)	0.03 (0.781)	0.04 (0.690)	0.07 (0.194)
Proportion of TCM diagnosis and treatment cost								
Aged <60 years	9.55 (<0.001)	−0.09 (0.842)	1.17 (0.633)	−0.04 (0.951)	−1.05 (0.648)	0.87 (0.012)	−0.12 (0.621)	0.75 (<0.001)
Aged ≥60 years	149.04 (<0.001)	−8.49 (0.182)	29.01 (0.487)	6.39 (0.412)	−18.39 (0.618)	7.82 (0.069)	−2.10 (0.533)	5.72 (0.006)
Proportion of discharged patients treated mainly with TCM								
Aged <60 years	78.08 (<0.001)	2.47 (0.001)	−16.36 (0.004)	−3.41 (<0.001)	20.87 (<0.001)	0.91 (0.037)	−0.94 (0.013)	−0.03 (0.864)
Aged ≥60 years	84.39 (<0.001)	1.35 (0.193)	−6.46 (0.409)	−2.93 (0.012)	25.60 (<0.001)	1.66 (0.005)	−1.58 (0.002)	0.07 (0.789)

#### 3.5.2 Interrupted time series analysis of cost structure indicators by age subgroup

In the group under 60 years of age, during the DRG reform, the average TCM drug cost per case decreased instantaneously by CNY 200.96 (*p* = 0.002), with a trend change (*β_3_*) of −18.40 (*p* = 0.040) before and after the reform. After the reform, the average TCM drug cost per case increased by an average of CNY 19.19 per half month (*p* < 0.001), while changes in average TCM preparation cost per case and average TCM diagnosis and treatment cost per case were not statistically significant. Upon the implementation of the Value-Based payment reform for therapeutic effects, the average TCM preparation cost per case decreased instantaneously by CNY 81.06 (*p* = 0.042), and the average TCM drug cost per case decreased instantaneously by CNY 121.63 (*p* = 0.012), with *β_5_* values of 7.99 (*p* = 0.033) and −13.30 (*p* = 0.004), respectively. After the reform, the average TCM preparation cost per case increased by CNY 10.19 per half month (*p* < 0.001), the average TCM drug cost per case increased by an average of CNY 5.89 per half month on average (*p* = 0.007), and the average TCM diagnosis and treatment cost per case increased by CNY 104.54 every half month on average (*p* < 0.001).

**Figure 5:**
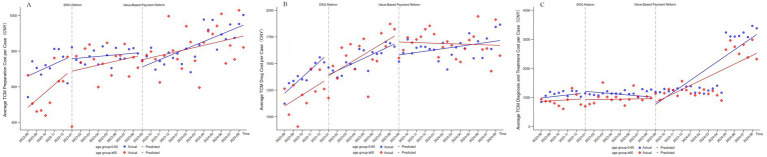
Interrupted time series trend chart of cost structure indicators by age subgroup. Three line charts compare actual and predicted costs over time in Chinese yuan for two age groups—under sixty and sixty or older—in average TCM preparation cost per case **(A)**, average TCM drug cost per case **(B)**, and average TCM diagnosis and treatment cost per case **(C)**. Each chart features vertical lines marking DRG Reform and Value-Based Payment Reform periods, with visible trends and fluctuations differentiated by age group and cost type.

In the group aged 60 years and above, during the DRG reform, there were no significant changes in the average TCM preparation cost per case or the average TCM diagnosis and treatment cost per case, while the average TCM drug cost per case increased by an average of CNY 24.94 per half month after the reform (*p* = 0.001). Upon the implementation of the Value-Based payment reform for therapeutic effects, only the average TCM diagnosis and treatment cost per case increased by an average of CNY 73.25 per half month after the reform (*p* < 0.001), with no significant changes in the average TCM preparation cost per case or the average TCM drug cost per case (Figure 5; Table 4).

#### 3.5.3 Interrupted time series analysis of TCM characteristics indicators by age subgroup `

In the group under 60 years of age, during the DRG reform, the *β_2_* values for the proportion of preparation cost in total drug revenue, the proportion of TCM drug cost in total drug revenue, and the proportion of discharged patients treated mainly with TCM were 9.28% (*p* < 0.001), 7.06% (*p* = 0.035), and −16.36% (*p* = 0.004), the change in the trend of the proportion of discharged patients treated mainly with TCM before and after the reform (*β_3_*) was −3.41% (*p* < 0.001) respectively. After the reform, only the proportion of discharged patients treated mainly with TCM decreased by an average of 0.94% per half month (*p* = 0.013). Upon the implementation of the Value-Based payment reform for therapeutic effects, the proportion of discharged patients treated mainly with TCM increased instantaneously by 20.87% (*p* < 0.001), with a *β_5_* value of 0.91% (*p* = 0.037). The proportion of preparation cost in total drug revenue increased by an average of 0.46% per half month (*p* = 0.008), increased by an average of 0.58% per half-month (*p* = 0.050), and the proportion of TCM diagnosis and treatment cost increased by an average of 0.75% per half month (*p* < 0.001).

**Figure 6:**
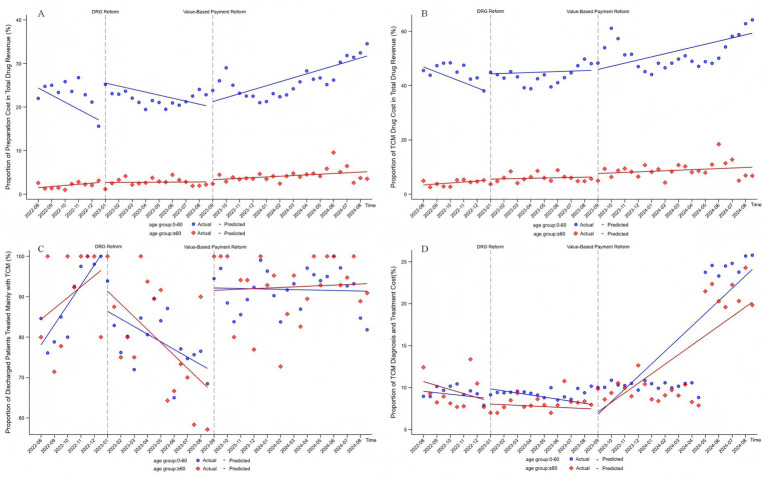
Interrupted time series trend chart of TCM characteristic indicators by age subgroup. Grouped image of four scatter plots with trend lines, labeled A through D, each showing time series data segmented by two age groups: under sixty and over sixty, using blue dots and lines for younger and red diamonds and lines for older. Vertical dashed lines indicate timing of DRG Reform and Value-Based Payment Reform. Panel **A** shows proportion of preparation cost in total drug revenue, panel **B** shows proportion of TCM drug cost in total drug revenue, panel **C** shows proportion of discharged patients treated mainly with TCM, and panel **D** shows proportion of TCM diagnosis and treatment cost.

In the group aged 60 years and above, during the DRG reform, there were no statistically significant instantaneous changes in the proportion of preparation cost in total drug revenue, the proportion of TCM drug cost in total drug revenue, or the proportion of TCM diagnosis and treatment cost, while the proportion of discharged patients treated mainly with TCM decreased by an average of 1.58% per half month after the reform (*p* = 0.002). Upon the implementation of the Value-Based payment reform for therapeutic effects, there were no statistically significant instantaneous changes in the proportion of preparation cost in total drug revenue, proportion of TCM drug cost in total drug revenue, or the proportion of TCM diagnosis and treatment cost. Only the proportion of discharged patients treated mainly with TCM increased by 25.60% (*p* < 0.001), the change in the trend before and after the reform (*β_5_*) was 1.66% (*p* = 0.005). However, the proportion of preparation cost in total drug revenue increased by an average of 0.08% per half month on average (*p* = 0.045), and the proportion of TCM diagnosis and treatment cost increased by an average of 0.57% per half month on average (*p* < 0.001; Figure 6; Table 4).

## 4 Discussion

This retrospective analysis of 5,304 cases across three phases (the pre-DRG reform, the post-DRG reform, and the post-Value-Based payment reform) revealed the dual impacts of DRG payment reform and Value-Based payment reform on medical service models, particularly highlighting the pivotal role of Value-Based payment in unleashing the value of TCM services. The results demonstrate the distinct trends in key variables across the three phases.

### 4.1 Analysis of the dual-track effects of DRG payment and value-based payment

As a medical insurance payment tool, DRG helps control the unreasonable growth of medical expenses and improve the quality of medical services (38). After the implementation of DRG reform, average inpatient cost decreased instantaneously by CNY 1,131.27, and average length of stay decreased instantaneously by 1.69 days. This trend was consistent with the objectives of DRG payment to control hospitalization expenses and optimize the allocation of medical resources (39, 40). However,in the long term, the growth rate of medical expenses was not fully curbed, with the average inpatient cost continuing to rise at CNY 62.88 per half-month. This phenomenon was closely associated with the fact that China’s DRG payment standards have not yet fully reflected the value of the TCM-specific services (39).

Starting from 2019, China piloted the Value-Based payment for selected TCM-dominant diseases in Liuzhou, Shanghai, Hunan, and other regions. This policy introduced a point substitution mechanism between TCM conservative treatment and Western medicine surgical treatment for the same disease based on the DRG point-based payment, which effectively reduces the upward trend of medical expenses after DRG reform (27). The growth rate of average inpatient cost decreased by another 8.12 yuan, stabilizing at CNY 54.76 per half-month post-Value-Based payment reform, the average length of stay showed a downward trend compared with that before the DRG reform. It indicated that on the basis of DRG cost control, this policy may further optimize the diagnosis and treatment process and better meet the demand for TCM services (27, 40).

### 4.2 From cost containment to outcome-based value realization

Although the DRG reform has yielded sound effects on cost containment, its core design compels medical institutions to take initiative in cost control under bundled payment. Fundamentally a cost accounting system rooted in Western medical clinical pathways, DRG fails to highlight the distinctive strengths of TCM services when applied to TCM-dominant conditions (41). Implementing the DRG reform for TCM-dominant diseases fails to highlight the uniqueness of the TCM services. In this study, during the initial stage of DRG reform, average TCM drug cost per case decreased instantaneously by 170.95 yuan, and the proportion of discharged patients treated mainly with TCM plummeted by 13.73%. These findings indicate the poor compatibility between TCM and the DRG system: traditional DRG grouping struggles to reflect the value of TCM’s personalized diagnosis and treatment, which restricts the development of the TCM characteristics (42). As a unique resource in China’s healthcare system, TCM has been receiving strong national support for its inheritance and innovation. Under the DRG reform, the rollout of Value-Based payment for TCM-dominant conditions adopts an innovative mechanism of equal payment for equivalent outcomes for the same disease, which ensures TCM services are fairly remunerated (16, 17). Following the reform, this study found significant increases in average TCM preparation cost per case, average TCM diagnosis and treatment cost per case, the proportion of preparation cost in total drug revenue, proportion of TCM drug cost in total drug revenue, and the proportion of TCM diagnosis and treatment cost. These results confirm that the value of TCM services has been better recognized, and TCM characteristics have been more fully exerted.

The Value-Based payment reform for TCM-dominant diseases not only addresses the undervaluation of TCM services under the DRG but also promotes the full manifestation of TCM’s unique strengths, facilitating a win-win situation for medical institutions, patients, and insurance systems (43). The essence of Value-Based healthcare lies in controlling medical expense growth, optimizing patient experience, and incentivizing efficient service provision through quality assessment systems and reward mechanisms (44), and this aligns with the goals of medical insurance payment reform and the inherent logic of Value-Based payment for TCM services (26). Currently, the value demonstration of TCM is still in development, with simplistic evaluation methods and incomplete systems (45). To ensure the its long-term sustainability, future efforts should focus on optimizing Value-Based payment standards to accurately reflect the value of the TCM-specific services while curbing the unreasonable expense growth.

First, we should utilize the guiding role of medical insurance policies to improve Value-Based payment standards. We should develop standardized management protocols for TCM inpatient medical records to ensure the fairness and sustainability of the payment system (46); establish multi-dimensional evaluation systems integrating patient health improvement, treatment efficacy, and service efficiency to optimize resource allocation.

Second, the coverage of diseases predominantly treated with TCM under Value-Based payment should be gradually expanded (40). In line with the principle of equal payment for equivalent outcomes for the same disease by either TCM or Western medicine (47, 48), we may select diseases featuring distinct advantages, mature clinical practice, reliable efficacy and safety to be included in the payment scheme (49).

Third, we will build a dynamic monitoring system for outcome evaluation, integrate clinical pathways into outcome-based assessment, and set up evidence-based evaluation criteria for TCM (26). Given the problems of dual TCM-Western medicine coding and merged coding existing in TCM hospitals under DRG (50), it is advisable to add TCM-dominant disease modules to DRG grouping and apply independent coding and pricing to TCM-specific therapies such as acupuncture and tuina.

Fourth, we should strengthen the communication between medical insurance authorities and healthcare institutions, and implement incentive mechanisms for physicians and departments. The insurance management departments should consider factors supporting TCM inheritance and innovation when setting hospital coefficients, providing appropriate preferential policies for TCM hospitals to explore the TCM-adapted payment reform. Meanwhile, with reference to the Operation Manual for Performance Appraisal of National Tertiary Public TCM Hospitals, we should clarify the management goals for TCM characteristics at the departmental level and improve the reward and punishment mechanisms for TCM service provision under the DRG system. In the departmental performance assessments, TCM-specific indicators such as the proportion of TCM prescriptions, the proportion of TCM non-pharmacological therapies, and the rate of TCM treatment should be incorporated. This will promote an integrated mechanism of performance appraisal and medical insurance payment, thereby advancing the high-quality development of TCM services.

### 4.3 Payment reform from the perspective of age heterogeneity

With the improvements in living standards and the impact of unhealthy lifestyles, the incidence of anorectal diseases has gradually shown a trend toward a younger population. To explore the sensitivity and responsiveness of different age groups to payment reform, this study further analyzed the reform effects in the group under 60 years of age and those aged 60 years and above, yielding important policy implications (51).

The results showed that the average inpatient cost in the group under 60 years of age decreased after the DRG reform, indicating a favorable cost-control effect. However, the average TCM drug cost per case and the proportion of discharged patients treated mainly with TCM decreased, which limited the development of TCM characteristics. In contrast, the group aged 60 years and above demonstrated a weaker overall response: only the proportion of discharged patients treated mainly with TCM decreased, while other indicators remained unchanged, suggesting that the existing DRG model failed to adequately adapt to the disease characteristics of the older adults. After the implementation of Value-Based payment reform, the group under 60 years of age exhibited a significant reduction in average inpatient cost and average length of stay, alongside marked increases in average TCM preparation cost per case, average TCM diagnosis and treatment cost per case, the proportion of preparation cost in total drug revenue, the proportion of TCM diagnosis and treatment cost, and the proportion of discharged patients treated mainly with TCM. These findings indicate that the group under 60 years of age was more sensitive to the Value-Based payment reform, likely due to their simpler disease spectrum, stronger recovery capacity, and higher efficiency in medical resource utilization. This suggests that policy reforms can effectively guide rational treatment-seeking behavior in this population. Conversely, the “dual insensitivity” pattern was observed among older adult patients: resource consumption indicators presented no significant variations, and TCM characteristic indicators achieved only marginal improvement. These differences are likely associated with the high prevalence of multiple comorbidities and complex treatment needs in the older adults. Studies have noted that age alone only slightly increases the average length of stay in the older adult patients without comorbidities or complications (52). Therefore, the payment reform should fully consider the uniqueness of older adult patients, by implementing measures such as optimizing treatment processes and improving service quality to further reduce their average length of stay and alleviate medical burdens.

From the perspective of age heterogeneity, Value-Based payment reform exerted differential effects on cost control and TCM service utilization among anorectal disease patients in different age groups, with more prominent impacts in the group under 60 years of age. Therefore, the refined design of payment policies should fully consider the age factor of patients to achieve more precise governance. When implementing Value-Based payment for TCM-dominant diseases, the age factor of patients should be fully considered, such as introducing age-disease interaction weight coefficients in DRG grouping and formulating differentiated policy measures for different age groups to achieve more refined and personalized governance and maximize the advantages of TCM (53). For the patients under 60 years of age, the payment policy should strive to consolidate and expand the reform achievements. In this study, the average inpatient cost of this group decreased and the average length of stay shortened after the implementation of the Value-Based payment.policy. Accordingly, the medical insurance payment mechanism can be further optimized to strengthen incentives. In line with the principle of equal payment for equivalent clinical outcomes for the same disease, policymakers may formulate improved payment standards for the patients under 60 years of age. This approach fully acknowledges the strong clinical response and sound cost-effectiveness of TCM services for this population, encouraging medical institutions to consistently deliver and upgrade TCM treatment regimens that excel in efficacy and operational efficiency. For the older adult patients, the payment policy should focus on resolving their special clinical and economic risks. The resource consumption indicators of the group aged 60 years and above in this study did not show significant improvement, indicating a tension between their disease complexity and the unified payment standard. It is recommended to systematically incorporate age and the severity of complications (such as the Charlson Comorbidity Index) as key adjustment factors in DRG grouping and weight setting (54), and consider establishing additional risk compensation channels for older adults and high-risk cases. At the same time, in the Value-Based payment reform, it is necessary to set efficacy evaluation dimensions and payment standards more in line with the diagnosis and treatment characteristics of the older adults. This mechanism accurately compensates medical institutions for excess resource consumption in the care of complex older adults, avoids cutbacks in essential services driven by economic concerns, and sustains a dynamic balance between cost control and quality assurance. Meanwhile, it enhances recognition of TCM service value and advances payment innovation, facilitating the expanded application and high-quality development of TCM services for TCM-dominant diseases.

### 4.4 Limitations and future directions

Although the design of this study is capable of identifying the effects of policy interventions to a certain extent, the following limitations remain: firstly, the absence of a parallel control group. Although ITS can identify the effects of DRG reform and Value-Based payment reform through multiple intervention nodes, a single-group before-and-after design still struggles to fully exclude the interference of time-dependent confounding factors, such as other concurrent healthcare reforms, institutional management adjustments, and changes in the disease spectrum. Compared to a multi-group ITS design involving an “intervention group–control group”, the ability to attribute policy effects is relatively limited. Second, there are limitations regarding the study sample and observation period. The data for this study were sourced from a single healthcare institution, which is highly specialized in TCM and has strong policy implementation capabilities; furthermore, as the region to which it belongs is a National Comprehensive Reform Demonstration Zone for TCM, it possesses a well-established supporting policy environment. For institutions where TCM characteristics are not prominent, technical expertise is weak, or systematic institutional support is lacking, the generalisability of the results may be somewhat limited. Furthermore, this study focuses solely on a few TCM-dominant anorectal conditions, such as mixed hemorrhoids and anal fistulas, and the observation period is relatively short, which limits its ability to reflect the long-term effects of policy implementation. Thirdly, the evaluation indicator system is not yet comprehensive. Existing indicators primarily revolve around medical costs and resource consumption; whilst they cover indicators specific to TCM, they do not systematically incorporate clinical and health outcome indicators such as therapeutic efficacy and patient satisfaction. Consequently, the dimensions for assessing the value of the policy remain to be expanded.

Future research should be expanded in the following areas: Firstly, the sample scope should be broadened to include medical institutions of different levels across various regions, as well as a wider range of conditions where TCM holds a comparative advantage. The observation period should be extended to enable a comprehensive, multi-dimensional assessment of the reform’s impact—covering clinical outcomes, service efficiency and patient experience—thereby validating the generalisability and stability of the research conclusions; secondly, building upon existing analyses of age subgroups, further stratification factors such as hospital tier and disease severity could be introduced to systematically examine the heterogeneous effects of Value-Based payment across different contexts, thereby providing more robust evidence to support the precision and scientific rigour of TCM payment reform.

## 5 Conclusion

This study explored the impacts of the dual policies (the DRG payment reform and the Value-Based payment reform) on the medical resource consumption and TCM characteristic indicators among the patients with anorectal diseases. The results indicate that the Value-Based payment effectively promotes the development of TCM advantages while retaining the cost-control benefits of the DRG reform. The subgroup analysis by age revealed more prominent policy effects in the populations under 60 years of age, suggesting that the implementation of the Value-Based payment requires adjustments tailored to specific patient subgroups. Due to the policy lag effects, the long-term impacts of such dual payment policies on the TCM-dominant diseases warrant further investigation. This study provides methodological evidence for the integration of the Value-Based healthcare concept with TCM payment from a practical perspective, which is conducive to constructing a scientific and rational TCM payment system, laying a foundation for the continuous optimization of relevant policies, and thus promoting the high-quality development of TCM services.

## Data Availability

The raw data supporting the conclusions of this article will be made available by the authors, without undue reservation.

## References

[1] Zhang S Zhang J Kong X. A study on the selection of conditions for which traditional Chinese medicine has a comparative advantage, the current status of medical insurance reimbursement methods, and strategies for promotion. Chin Hosp Manag. (2026) 46:61–4.

[2] Yang H Cui Z Wang M. Distribution and characteristics of conditions for which traditional Chinese medicine has a comparative advantage in China. J Tradit Chin Med. (2012) 53:285–7. doi: 10.13288/j.11-2166/r.2012.04.001

[3] Jiang W Zhang H Sui N. Epidemiological survey of common anorectal diseases among urban residents in China. Chin J Public Health. (2016) 32:1293–6. doi: 10.11847/zgggws2016-32-10-01

[4] Cheng Y Wu Y Li W. Progress in epidemiological surveys of anorectal diseases in China. Chin J Anorectal Dis. (2022) 42:74–6. doi: 10.3969/j.issn.1000-1174.2022.06.033

[5] Lai D Wu Y. The effect of Chinese herbal sitz baths combined with acupuncture at the eight Liao points on patients after surgery for mixed haemorrhoids. Chin Foreign Med Res. (2025) 23:50–3. doi: 10.14033/j.cnki.cfmr.2025.10.014

[6] Liu A. A Study on the Efficacy of Acupuncture Combined with Chinese Herbal Sitz Baths in Treating Postoperative Pain in Anal and Rectal Diseases. Sichuan: North Sichuan Medical College (2024).

[7] Hu X Chen H Peng J. Progress in the external treatment of anal margin oedema following surgery for mixed haemorrhoids using traditional Chinese medicine. Guangming Tradit Chin Med. (2024) 39:821–4. doi: 10.3969/j.issn.1003-8914.2024.04.058

[8] Quentin WG A Scheller-Kreinsen D Busse R. DRG-type hospital payment in Germany: the G-DRG system. Euro Observer. (2010) 12:4–6.

[9] Vera DEA Gillett S. AR-DRG Australia Refined Diagnosis Related Groups Version 8.0 Definition Manual Volume 1. Sydney: Australian Consortium for Classification Development (2014).

[10] (InEK). G-DRG German Diagnosis Related Groups Version 2012 Definitionshandbuch Band 1. Siegburg: InEK (2011).

[11] Zheng B. Reform of medical insurance payment methods in China: current status, challenges and trends. People’s Forum. (2025) 5:44–50.

[12] Yuan L Xiang Y Shen J. The effectiveness of DRG application in the medical management of traditional Chinese medicine hospitals. J Tradit Chin Med Manag. (2022) 30:68–70. doi: 10.16690/j.cnki.1007-9203.2022.11.024

[13] Liu D Li Y Guo Y. Analysis of the application of DRG in medical management at traditional Chinese medicine hospitals. Chin Hosp Manag. (2020) 40:44–7.

[14] Xu X. A Study on Inpatient Costs Based on the Principles of Diagnosis-Related Groups (DRG): A Case Study of Haemorrhoids (Mixed Haemorrhoids) at S Traditional Chinese Medicine Hospital. China: Shandong University (2020).

[15] Xie J Zhang Q Wang Z. Reflections on payment methods and standards for traditional Chinese medicine services under medical insurance. China Med Insur. (2017) 7:51–3. doi: 10.19546/j.issn.1674-3830.2017.7.012

[16] State Administration of Traditional Chinese Medicine (2021) Guidelines on Medical Insurance Support for the Inheritance and Innovative Development of Traditional Chinese Medicine (Medical Insurance Letter [2021] No. 229). Available online at: https://www.nhsa.gov.cn/art/2021/12/31/art_104_7721.html (Accessed February 24, 2026).

[17] Hunan Provincial Medical Security Bureau (2023) Notice on the Implementation of Value-Based Payment for TCM-Advantaged Diseases within Regional DRG Payment (Trial) (Xiang Yibao Fa [2023] No. 31). Available online at: http://ybj.hunan.gov.cn/ybj/first113541/firstF/f2113606/202308/t20230802_29451506.html (Accessed February 24, 2026).

[18] Liao ZY. The concept and policy framework of value-based payment for traditional Chinese medicine in the DRG era. China Hum Resour Soc Secur. (2021) 5:59. doi: 10.3969/j.issn.1674-9111.2021.05.032

[19] Porter ME. What is value in health care? N Engl J Med. (2010) 363:2477–81. doi: 10.1056/NEJMp1011024, 21142528

[20] Institute of Medicine Committee on Quality of Health Care in America. Crossing the Quality Chasm: A New Health System for the 21st Century. Washington, DC: National Academies Press (US) (2001).

[21] Guo R Zeng D Hu YH. Approaches and methods for evaluating the advantages of traditional Chinese medicine from a value-based healthcare perspective. Sci Technol Rev. (2023) 41:14–21. doi: 10.3981/j.issn.1000-7857.2023.14.002

[22] Li DN Guo H Niu L Yin QS Zhang YJ Zhuang PW. Clinical value-oriented research paradigm regarding the inheritance and innovative development of TCM-dominant diseases. Chinese Herbal Med. (2023) 15:476–84. doi: 10.1016/j.chmed.2023.09.002, 38094019 PMC10715888

[23] Jin C Wang H Sun H. The concept, practice and implementation pathways of value-based healthcare. Health Econ Res. (2019) 36:6–8. doi: 10.14055/j.cnki.33-1056/f.2019.02.002

[24] Fang L Wang H. A study on the practice of ‘value-based healthcare’ in the United States and its implications. Health Soft Sci. (2019) 33:21–6. doi: 10.3969/j.issn.1003-2800.2019.12.005

[25] Gao P Liu Y. The concept of value-based healthcare and related research progress. Chinese J Healthcare Manag Sci. (2022) 12:57–62.

[26] Huang C Shen L Yang Y. A discussion on the effectiveness of value-oriented medical insurance payment policies: an empirical analysis based on traditional Chinese medicine’s advantageous disease categories in Liuzhou City. Health Econ Res. (2022) 39:37–41. doi: 10.14055/j.cnki.33-1056/f.2022.05.009

[27] Liao C Qin J. A study on the implementation effects of value-based payment for traditional Chinese medicine: a case study of mixed haemorrhoids. J Nanjing University of Chinese Med (Soc Sci Edition). (2024) 25:225–30. doi: 10.20060/j.cnki.ISSN1009-3222.2024.0225

[28] Mi L Zhou L Sun L. Application and effectiveness evaluation of value-based payment in the innovative development of traditional Chinese medicine. Chinese Public Health Manag. (2025) 41:804–8. doi: 10.19568/j.cnki.23-1318.2025.06.0008

[29] Liu C. A Study on the Evaluation of the Implementation Effectiveness of Value-Based Payment for Conditions with TCM Advantages: A Case Study of City C, Hunan Province. Hunan: Hunan University of Chinese Medicine (2025).

[30] Mao Y Zhou L. Implementation outcomes of value-based payment policies for traditional Chinese medicine under the DRG system. Health Develop Policy Res. (2025) 28:715–23. doi: 10.12458/HDPR.202509093

[31] Hu JZ H Feng S Li B. Application of comprehensive evaluation methods in the assessment of clinical efficacy of traditional Chinese medicine: a narrative review. Longhua J Tradit Chin Med. (2022) 5:25. doi: 10.21037/lcm-21-63

[32] Zhao FY Yue LP Xu PJ Conduit R Zhang WD Li YX . Harmonising methodological paradigms to more accurately evaluate personalised TCM interventions in standardised trials: introducing the TRIPLE-TCM trial framework. Ther Clin Risk Manag. (2025) 21:1521–34. doi: 10.2147/TCRM.S557457, 41216025 PMC12597255

[33] Yang J Li M Shen J. Experiences and insights from pilot schemes on payment reform for TCM-advantaged conditions. Health Econ Res. (2023) 40:33–8. doi: 10.14055/j.cnki.33-1056/f.2023.12.009

[34] Linden A. A matching framework to improve causal inference in interrupted time series analysis. J Eval Clin Pract. (2018) 24:408–15. doi: 10.1111/jep.12874, 29266646

[35] Xu X Ou C. Interrupted time series methods for evaluating the effectiveness of public health interventions and their applications. Chin J Health Stat. (2023) 40:41–4. doi: 10.11783/j.issn.1002-3674.2023.01.009

[36] Linden A. Conducting interrupted time-series analysis for single- and multiple-group comparisons. The Stata J: Promoting communications on statistics and Stata. (2015) 15:480–500. doi: 10.1177/1536867X1501500208

[37] Kutz A Gut L Ebrahimi F Wagner U Schuetz P Mueller B. Association of the Swiss Diagnosis-Related Group Reimbursement System with length of stay, mortality, and readmission rates in hospitalized adult patients. JAMA Netw Open. (2019) 2:e188332. doi: 10.1001/jamanetworkopen.2018.8332, 30768196 PMC6484617

[38] Wang Y Sun Z Chen Y. A review of the progress in DRG research and practice in representative countries and its implications for China. Chin J Health Econ. (2021) 40:91–6.

[39] Zhang H Ding K Xie J. Exploration of a DRG payment scheme for TCM-advantaged conditions under ‘outcome-based payment’. Health Econ Res. (2021) 38:75–6. doi: 10.14055/j.cnki.33-1056/f.2021.12.021

[40] Yu W Li J Zhong L. Analysis of the effects of the reform of outcome-based payment for TCM-advantaged diseases. Health Econ Res. (2025) 42:23–7. doi: 10.14055/j.cnki.33-1056/f.2025.04.018

[41] Liang L Gao H Feng Q. A comparative study on the current status of DRG development at home and abroad. Health Soft Sci. (2020) 34:65–9.

[42] Zhu X Tian K Fang P. Discussion on several issues and countermeasures regarding DRG payment in traditional Chinese medicine hospitals in China. Chin Hosp. (2022) 26:10–2. doi: 10.19660/j.issn.1671-0592.2022.5.03

[43] Su F Xiao C. A study on the implementation effects and optimisation strategies of the value-based payment policy for traditional Chinese medicine: a comparison based on typical regions. Hunan J Tradit Chin Med. (2025) 41:188–92. doi: 10.16808/j.cnki.issn1003-7705.2025.01.038

[44] Wang Y Feng R. A conceptual framework for the realisation of value-based healthcare through reform of medical insurance payment methods. Chin J Health Econ. (2022) 41:21–3.

[45] Wang Y. A Study on the Analysis of the Connotation and System Construction of Personalised Efficacy Evaluation in Traditional Chinese Medicine Clinical Practice. Fuji: Fujian University of Traditional Chinese Medicine (2018).

[46] Gao X Liu Z Jiang X. A discussion on the implementation effectiveness of the integrated traditional Chinese and Western medicine DRG payment model in traditional Chinese medicine hospitals. Mod Hosp. (2025) 25:910–912+916.

[47] Zhang L Li Q Li X. Practice and reflections on payment reform incorporating elements of traditional Chinese medicine from a value perspective. Chin Health Econ. (2023) 42:1–5.

[48] Liu Y Zhou D Lin L. An exploration of the implementation of TCM DRG payment policies based on the principle of ‘same disease, same price’. Chin Hosp Manag. (2025) 45:16–9.

[49] Wu Q Zhang Z Feng D. The impact of DRG reform on traditional Chinese medicine services in TCM hospitals: a discontinuous time series analysis. Chin Health Serv Manag. (2025) 42:74–8.

[50] Ma Z Luan R Xiao P. Discussion on common issues in DRG disease coding in key departments of traditional Chinese medicine hospitals. Chin Health Econ. (2020) 39:93–5. doi: 10.7664/CHE20201222

[51] Chen YJ Zhang XY Tang X Yan JQ Qian MC Ying XH. How have costs, length of stay and quality of care changed for inpatients of different age groups following the implementation of the new diagnosis-related group (DRG) payment reform in China? A interrupted time series analysis. BMC Health Serv Res. (2023) 23:160. doi: 10.1186/s12913-023-09109-z, 36793088 PMC9933283

[52] DesHarnais SI Chesney JD Fleming ST. Should DRG grouping be based on age? Health. (1988) 26:124–31. 10.1097/00005650-198802000-00004 3123815

[53] Dai Z Liu L Man X. Analysis of the population benefiting from treatment costs for priority conditions in Beijing’s traditional Chinese medicine system based on ‘SHA 2011’. Zhongguo Wei Sheng Jing Ji. (2011) 43:45–8.

[54] Keohane LM Stevenson DG Stewart L Thapa S Freed S Buntin MB. Risk adjustment for Medicaid participation in Medicare advantage plans. Am J Manag Care. (2020) 26:e258–63. doi: 10.37765/ajmc.2020.44076, 32835468 PMC7864213

